# Fabrication and Characterization of Humidity Sensors Based on Graphene Oxide–PEDOT:PSS Composites on a Flexible Substrate

**DOI:** 10.3390/mi11020148

**Published:** 2020-01-29

**Authors:** Francisco J. Romero, Almudena Rivadeneyra, Markus Becherer, Diego P. Morales, Noel Rodríguez

**Affiliations:** 1 Pervasive Electronics Advanced Research Laboratory, University of Granada, 18071 Granada, Spain; arivadeneyra@ugr.es; 2 Department of Electronics and Computer Technology, University of Granada, 18071 Granada, Spain; diegopm@ugr.es; 3 Chair of Nanoelectronics, Technical University of Munich, 80333 München, Germany; markus.becherer@tum.de; 4 Biochemistry and Electronics as Sensing Technologies Group, University of Granada, 18071 Granada, Spain

**Keywords:** flexible electronics, graphene oxide, humidity, PEDOT:PSS, screen-printing, sensor

## Abstract

In this paper, we present a simple, fast, and cost-effective method for the large-scale fabrication of high-sensitivity humidity sensors on flexible substrates. These sensors consist of a micro screen-printed capacitive structure upon which a sensitive layer is deposited. We studied two different structures and three different sensing materials by modifying the concentration of poly(3,4-ethylenedioxythiophene)/polystyrene sulfonate (PEDOT:PSS) in a graphene oxide (GO) solution. The results show that the aggregation of the PEDOT:PSS to the GO can modify its electrical properties, boosting the performance of the capacitive sensors in terms of both resistive losses and sensitivity to relative humidity (RH) changes. Thus, in an area less than 30 mm^2^, the GO/PEDOT:PSS-based sensors can achieve a sensitivity much higher (1.22 nF/%RH at 1 kHz) than other similar sensors presented in the literature which, together with their good thermal stability, time response, and performance over bending, demonstrates that the manufacturing approach described in this work paves the way for the mass production of flexible humidity sensors in an inexpensive way.

## 1. Introduction

Over the last few years, flexible electronics attracted more and more interest in diverse fields of science and technology. Thus, it is now a rapidly developing field of research boosted by the recent advances in two transversal fields: materials for flexible electronics and the compatible fabrication technologies [1]. This interest comes up in response to the challenges imposed by the new electronic applications of the Internet of things (IoT) era, where a trend toward ubiquitous sensing is becoming increasingly clear [2]. In this context, the number of sensing variables, whether environmental or biological, as well as the different ways they can be addressed, resulted in a wide range of studies, among which relative humidity (RH) sensors attracted notable attention given their importance for diverse processes and industries, such as food, biomedicine, and the living environment [3,4,5,6]. Numerous materials have been considered for the manufacturing of flexible RH sensors, such as carbon nanotubes (CNTs) [7], silicon nanosheets (SiNSs) [8], metal–organic frameworks (MOFs) [9], polymers [10], or oxides [11,12]. However, although some of these sensors reported auspicious results, a technology that enables a cost-effective manufacturing of small flexible RH sensors with a full range of sensitivity, low thermal drift, and fast response is still being sought [13]. 

In this work, we studied two promising materials for this purpose, graphene oxide (GO) and poly(3,4-ethylenedioxythiophene)/polystyrene sulfonate (PEDOT:PSS). On one hand, GO is a perfect candidate to act as a sensing material in RH sensors since, due to its high hydrophilicity, it absorbs the water molecules into its structure, thereby modifying some of its properties, such as the electrical conductivity or the dielectric behavior [14]. On the other hand, PEDOT:PSS is widely used in combination with a large variety of organic materials in order to tune its inherent properties, such as conductivity, dielectric constant, or flexibility [15,16]. Following this direction, we present a cost-effective approach for the fabrication of flexible RH sensors using a GO/PEDOT:PSS composite as a sensitive layer. We opted for an in-plane capacitive structure consisting of several Ag printed interdigitated electrodes (IDEs), since this configuration allows the fabrication of sensors with lower thickness and smaller distances between electrodes than other conventional technologies [17]. Moreover, we explored not only the influence of the PEDOT:PSS concentration, but also the capacitive structure itself in terms of sensitivity to humidity changes, frequency response, and losses. Additionally, we also performed tests of thermal stability, time response, and mechanical stress. 

This work is structured as follows: following this introduction, Section 2 summarizes the materials used for the fabrication of the sensors, together with the methodologies for their characterization. Section 3 presents both structural and electrical results for two different IDE structures using GO and a hybrid GO/PEDOT:PSS composite as a sensitive layer. Finally, the main conclusions are drawn in Section 4.

## 2. Materials and Methods 

### 2.1. Materials

Transparent and flexible films intended for water-based inks with a thickness of 160 µm (from ColorGATE Digital Output Solutions GmbH, Hannover, Germany) were used as substrate for the fabrication of the RH capacitive sensors. GO colloid with a concentration of 4 mg/mL (0.4 wt%) was prepared following a modified version of Hummers and Offerman’s method [18]. The PEDOT:PSS dispersion used in this work was obtained from Heraeus Holding GmbH (Hanau, Germany, product name: CLEVIOS™ P VP AI 4083). Conductive patterns were achieved using a silver-based screen printable ink (LOCTITE^®^ ECI 1010 E&C from Henkel AG, Düsseldorf, Germany).

### 2.2. Fabrication of the RH Sensors

**Figure 1:**
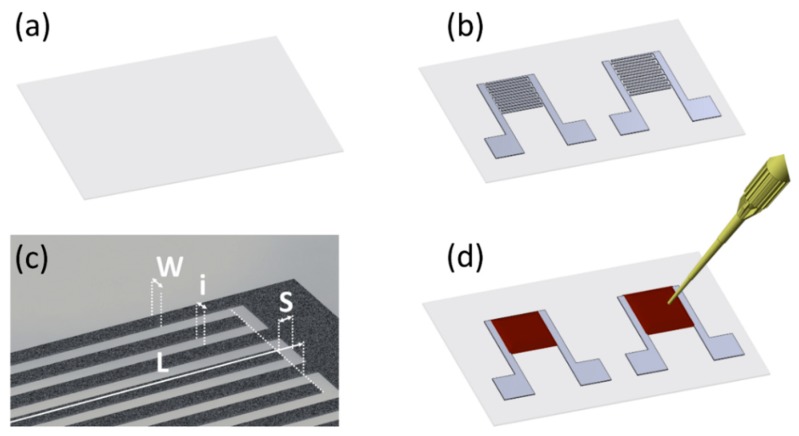
Schematic representation of the fabrication process of the relative humidity (RH) sensors. (**a**) Flexible transparent substrate (thickness: 160 μm). (**b**) Capacitive interdigitated electrode (IDE) structure screen-printed on the substrate. (**c**) Dimensions of the interdigitally arranged electrodes (*W*: width, *i*: interspacing, *L*: length, *S*: spacing). (**d**) Sensitive layer drop-casted on top of the IDE structure.

The manufacturing procedure of the capacitive humidity sensors presented in this work is schematized in Figure 1. Firstly, two interdigitally arranged electrodes (IDE) were printed on the flexible substrate (Figure 1a) using a manual screen-printer (from Siebdruck-Versand, Magdeburg, Germany) with a mesh of 90 nylon threads per centimeter (T/cm). Two different IDE structures were considered, as shown in Figure 1b. Both of them share a similar area (23 mm^2^ and 27 mm^2^) but different dimensions. Each one of these capacitive structures follows the pattern indicated in Figure 1c, where *W* is the width of finger, *i* is the interspacing between fingers, *S* is the spacing between electrodes, and *L* is the length of the finger excluding the separation; each one of these electrodes consists of N fingers (2 × N electrodes in the complete sensing structure). The specific dimensions used for each capacitive structure can be found in Table 1. 

After the screen-printing process, the samples were dried using a UF55 oven (from Memmert GmbH + Co. KG, Schwabach, Germany) at 120 °C for 15 min, as recommended by the manufacturer. Then, three different sensitive layers were considered: GO and GO/PEDOT:PSS at two different concentrations of PEDOT:PSS (10% and 20%). For that, 50 µL of these three different solutions were drop-casted using a micropipette on the IDE structures as shown in Figure 1d. Finally, the samples were left standing overnight to remove the excess of water. A real view of one of these sensors can be seen in Figure S1 (Supplementary Materials).

### 2.3. Characterization

Fourier-transform infrared spectroscopy (FTIR) was performed using an ALPHA II FTIR spectrophotometer (from Bruker Corporation, Billerica, MA, USA). Optical microscope images were obtained with a ZEISS Axioscope 5 microscope and analyzed with the ZEN Core software (both from Carl Zeiss AG, Oberkochen, Germany). Scanning electron microscope (SEM) images were recorded using an Auriga FIB-FESEM microscope (from Carl Zeiss AG, Oberkochen, Germany). The thickness of the samples was acquired using a DekTak XT contact profilometer (from Bruker Corporation, Billerica, MA, USA) at a stylus force and a scan resolution of 1 mg and 0.33 µm, respectively. The sheet resistances were measured using the four-point method at a constant current of 100 μA with a probe head from Jandel connected to a B2901A source measuring unit (from Keysight Technologies, Inc., St. Rose, CA, USA). The performance of the capacitive humidity sensors was studied using the climate chamber VCL4006 (from Vötsch Industrietechnik GmbH, Balingen, Germany), together with the impedance analyzer 4294A (from Keysight Technologies, Inc., St. Rose, CA, USA). The impedance of the samples as a function of the frequency was measured for each value of temperature and humidity using an excitation signal of 500 mV. A custom bending set-up was employed to perform the mechanical stress tests using a PD4-N5918M420 stepper motor, together with a GPLE60 precision planetary gear (from Nanotec Electronic GmbH & Co. KG, Feldkirchen, Germany). The whole measurement set-up was automated using the software LabView 2017 (from National Instruments Corporation, Austin, TX, USA).

## 3. Results and Discussion

### 3.1. Structural Properties

Before the deposition of the sensitive layer, both capacitive IDE structures were analyzed under the microscope with the purpose of determining whether the pattern was properly transferred to the thin-film substrate without any short-circuit, as well as to establish a comparison between the desired dimensions of the IDE structure and those achieved with the screen-printing process.

**Figure 2:**
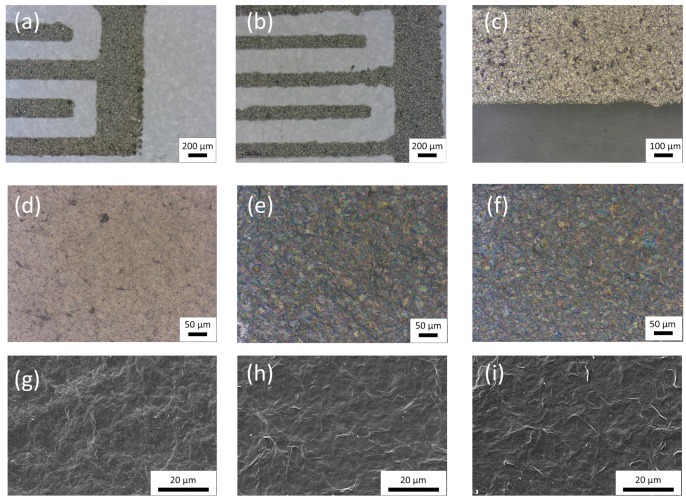
Optical microscope images: (**a**) screen-printed layout 1 (scale bar: 200 μm); (**b**) screen-printed layout 2 (scale bar: 200 μm); (**c**) Ag-based conductive ink screen-printed on the flexible substrate (scale bar: 100 μm); (**d**) graphene oxide (GO) sensitive layer (scale bar: 50 μm); (**e**) GO/poly(3,4-ethylenedioxythiophene)/polystyrene sulfonate (PEDOT:PSS) sensitive layer at 10% concentration (scale bar: 50 μm); (**f**) GO/PEDOT:PSS sensitive layer at 20% concentration (scale bar: 50 μm). SEM images: (**g**) GO sensitive layer (scale bar: 20 μm); (**h**) GO/PEDOT:PSS sensitive layer at 10% concentration (scale bar: 20 μm); (**i**) GO/PEDOT:PSS sensitive layer at 20% concentration (scale bar: 20 μm).

Figure 2a,b show a partial view of the two different patterns used in this work, so-called layout 1 and 2, respectively, in Table 1. At a glance it can be noted that smaller fingers resulted in worse resolution, since this was limited by the mesh of the screen mask [19]. Concretely, the real dimensions for both layouts are specified in Table 2, where errors represent the standard deviation of the measurements obtained for different fingers and samples. An image at higher magnification of one of these silver-based patterns is shown in Figure 2c, where the sandy texture as a consequence of the irregular flake structure of the Ag-ink can be noticed [20]. The average thickness of these patterns obtained through stylus profilometry was ~3 µm and their sheet resistance was 114 ± 11 mΩ/sq.

On the other hand, once the sensitive layers were deposited and dried on top of the IDE structures, they were also analyzed under microscope. In the case of the GO (Figure 2d), optical images show an uniform layer with a smooth surface and a few craters, which preserved the brown color of the aqueous solution of GO [21,22,23]. However, when the PEDOT:PSS was added to the GO solution, the dispersion turned dark blue, as seen in Figure 2e,f, since this is the hallmark color of PEDOT [24]. Moreover, as observed in SEM images (Figure 2g–i), the increase in PEDOT:PSS concentration yielded an increase of the roughness of the surface as a consequence of the PEDOT:PSS structure [25,26,27]. Experiments of the surface profilometry indicated an average sensitive layer thickness of around 3.5 µm.

**Figure 3:**
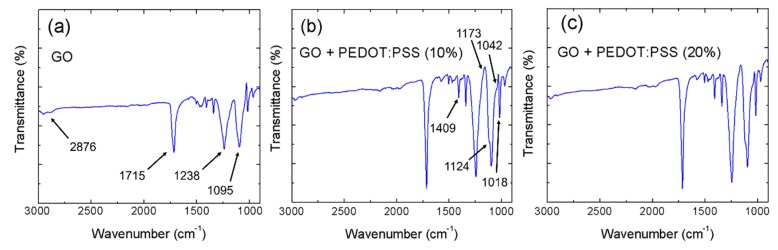
Fourier-transform infrared (FTIR) characterizations: (**a**) GO; (**b**) GO/PEDOT:PSS (10%); (**c**) GO/PEDOT:PSS (20%).

To further study the changes in the hybrid GO/PEDOT:PSS composites, the three sensitive layers were investigated using FTIR spectroscopy, whose results are presented in Figure 3. Firstly, starting with the GO spectrum (Figure 3a), three main peaks with a similar intensity could be clearly identified. These peaks indicated the high degree of oxidation of the carbon bonds, since they were associated with ether (C-O-C, 1095 cm^−1^), epoxy or alkoxy bonds (ν(C–O), 1238 cm^−1^), and carbonyl groups (C=O, 1715 cm^−1^) [28,29,30]. Moreover, a peak arising from the C-H stretching vibration at 2876 cm^−1^ was also present [31,32]. Furthermore, the addition of the PEDOT:PSS intensified the bands associated with the sulfur-containing groups remaining in the GO as a consequence of the H_2_SO_4_ oxidizing agent [18]. As it can be seen in Figure 3b, these peaks were located at 1409 cm^−1^, 1042 cm^−1^, and 1018 cm^−1^ and were associated with the S=O links [33,34]. Moreover, two new bands started appearing at 1173 cm^−1^ and 1124 cm^−1^, increasing with the content of PEDOT:PSS due to the SO_3_ stretching bands of the PSS structure [35]. Additionally, this increase in concentration also increased the ratio of the ν(C–O) bonds with respect the C=O ones, as seen in Figure 3c, since the first ones had a pervasive presence in the PEDOT structure.

### 3.2. Capacitive Humidity Sensor Characterization

**Figure 4:**
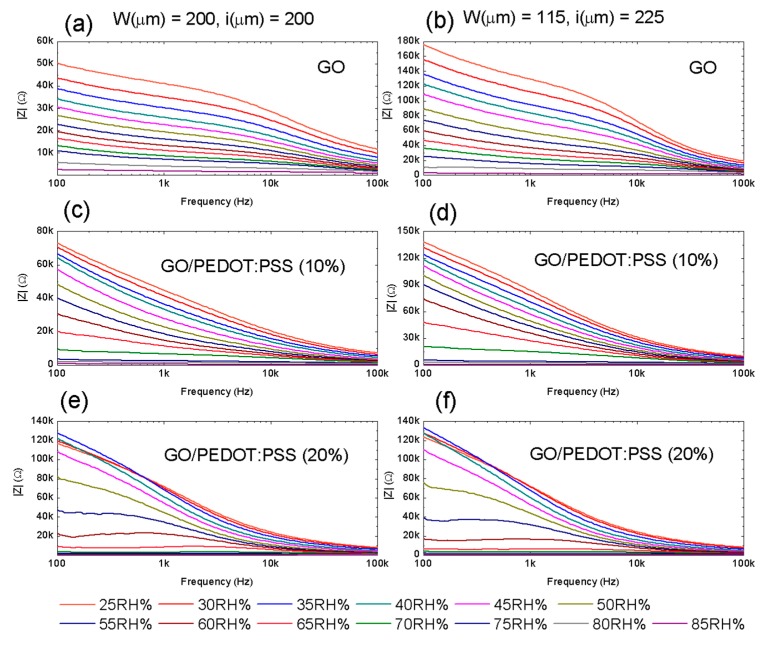
Absolute value of the impedance as a function of the frequency for the different values of RH. The left column presents the results obtained for layout 1 using the three different sensitive layers: (**a**) GO; (**c**) GO/PEDOT:PSS (10%); (**e**) GO/PEDOT:PSS (20%). Likewise, the right column presents the result of layout 2 for the same sensitive layers: (**b**) GO; (**d**) GO/PEDOT:PSS (10%); (**f**) GO/PEDOT:PSS (20%).

Firstly, the impedance of the flexible RH sensors (both real and imaginary parts) was measured as a function of the frequency modifying the relative humidity. The measurements were carried out at a constant temperature of 40 °C, since this operation point of the climate chamber allowed making use of the whole range of RH. Results of the absolute value of the impedance (|Z|) obtained for the different sensitive layers and IDE structures are collected in Figure 4. 

It can be noticed that the impedance modulus decreased as the frequency increased in all cases. Specifically, GO (Figure 4a,b) suffered a logarithmic decrease as a function of the frequency, as widely demonstrated in the literature. This indicates that, although both resistive and capacitive paths for the current-flow existed in the GO sensitive layer, the latter had a predominant role [36,37,38], as proven later. Moreover, the smaller width of electrodes and higher distance between them resulted in an increase in impedance modulus as a consequence of both a reduction of the interface between the GO film and the Ag electrode and an increase in equivalent resistance between electrodes. 

In the case of the hybrid GO/PEDOT:PSS composites, the decrease in impedance modulus with respect to the frequency was more abrupt, as can be observed for both concentrations of PEDOT:PSS and layouts (from Figure 4c–f). This effect is attributed to the high ratio of change of the PEDOT:PSS impedance when the frequency increases [39], which results in a double layer capacitance behavior [40]. However, it can also be observed that, in addition to changes in the frequency response of the capacitive sensors, the aggregation of PEDOT:PSS also induced changes in the response of the impedance to the RH. 

**Figure 5:**
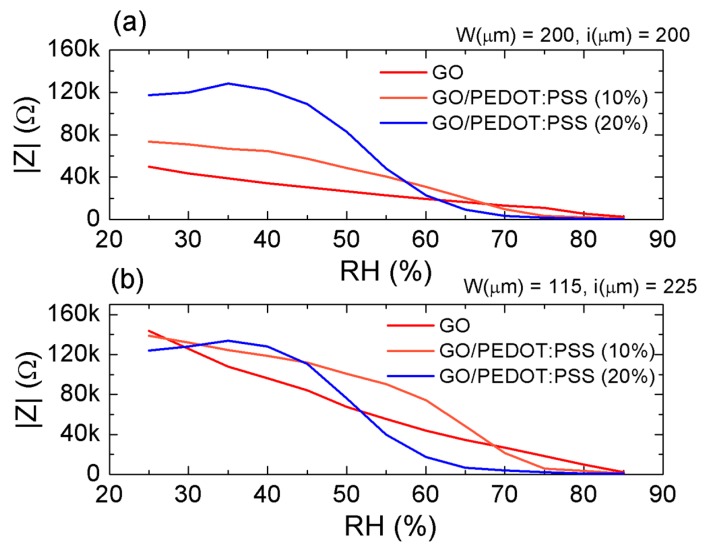
Absolute value of the impedance as a function of the relative humidity at a frequency of 100 Hz for both layout 1 (**a**) and layout 2 (**b**) using GO and the hybrid GO/PEDOT:PSS composites as sensitive layers.

To study this effect, the impedance modulus is plotted as a function of the RH at a fixed frequency for both IDE structures in Figure 5. Firstly, it can be noted that, as the content of PEDOT:PSS increased, the resistance of the sensitive layer became more influential than that corresponding to the interface between the sensitive layer and the Ag electrodes, which made the impedance of the wider fingers similar to that obtained with the *W* = 115 µm structure (blue curves in Figure 5). This might have been a consequence of the rougher surface morphology of the GO/PEDOT:PSS hybrid films, which contributed to a better contact at the interface electrode-sensitive film [41]. 

In any case, the impedance of both IDE structures using the same sensitive layer presented a similar behavior when measured as a function of the RH. On one hand, the GO sensitive films presented a highly linear decrease in impedance modulus for increasing values of RH. This sensitivity to humidity changes was due to the interaction of the hydrogen bonds of the water molecules with the surface of GO. With the increase in RH, a larger number of water molecules were physically adsorbed onto the GO film. These molecules, due to the electrostatic field, were ionized, forming hydronium ions (H_3_O^+^), thereby promoting the ionic conduction between fingers [11,38]. 

On the other hand, these results demonstrate that the aggregation of the PEDOT:PSS introduced changes in the impedance behavior. Thus, it can be noticed that, for the highest concentration of PEDOT:PSS (20%), at low levels of RH (<40%), the impedance modulus of the sensors suffered a slight increase, which indicated that the sensing effect of the PEDOT:PSS dominated the overall film impedance in this region. In this case, the increase in impedance modulus came from the interaction of both water and HSO_3_ molecules of the PSS chains. As the PSS absorbed the water molecules, the distance between adjacent PEDOT chains increased, leading to a decrease in electrical conduction [42]. Once the absorption of water molecules by the PSS chains saturated, the impedance decreased drastically due to the physisorption of the water molecules, which facilitated the ionic current and provides better current flow paths within the PEDOT structure [43]. This occurred up to ~70% RH, where the impedance modulus started decreasing more softly, since this point is defined as a maximum detection limit of the PEDOT [13]. Furthermore, a lower concentration of PEDOT:PSS led to a softer transition between these three different states, as can be observed in Figure 5. The linearity of the impedance modulus changes with respect to RH could also be partially restored by means of an increase in frequency at the cost of a lower ratio of change (see Figure S2, Supplementary Materials). 

**Figure 6:**
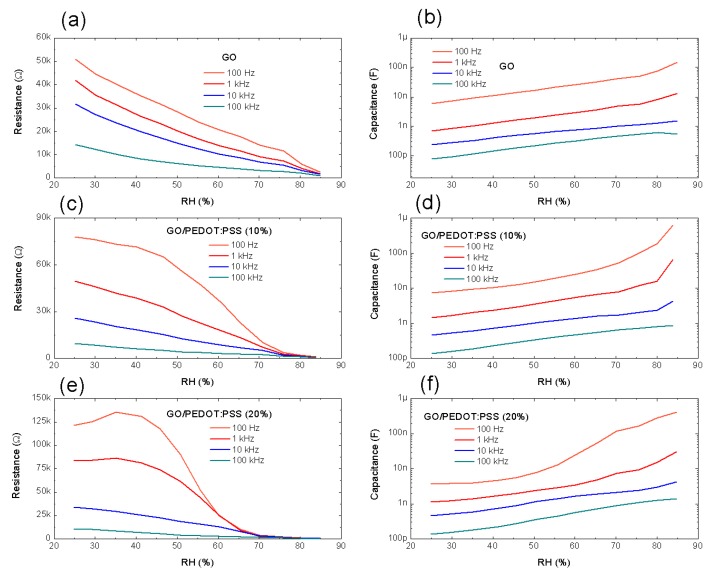
Equivalent parallel resistance and capacitance for layout 1 (*W* = 200 µm, *i* = 200 µm) at different frequencies using GO and the hybrid GO/PEDOT:PSS composites as sensitive layers; being (**a**,**b**) the results obtained for the GO layer, while (**c**,**d**) and (**e**,**f**) are the results associated to the GO/PEDOT:PSS (10%) and GO/PEDOT:PSS (20%) layers, respectively.

Once we demonstrated that the PEDOT:PSS was capable of modifying the impedance response of the sensors, we studied how these changes were translated into variations on its equivalent electrical circuit. An IDE planar capacitor, such as the ones presented in this work, can be modeled as an R_p_||Z_c_ association, where *R_p_* is the equivalent parallel resistance between electrodes (resistance of both electrode-sensitive layer interface and sensitive layer itself) and *Z_c_* is its reactance [13,38,44]. Following this model, we extracted the equivalent parallel resistance and capacitance as a function of the relative humidity at different frequencies. Figure 6 shows the results obtained for the sensors with dimensions *W* = 200 µm, *i* = 200 µm, while the results for the other layout can be consulted in Figure S3 (Supplementary Materials). 

As analyzed before, the changes in the impedance were ruled by the resistance behavior (see Figure 5). It can also be noticed that the increase in PEDOT:PSS involved lower losses due to the increase in equivalent *R_p_* (less shelf-discharge current) [45]. On the other hand, the capacitance increased as RH increased in all cases, which indicated that the water molecules adsorbed within the sensitive layer induced an increase in resulting dielectric permittivity [13]. Moreover, this increase also depended on the content of PEDOT:PSS, as seen in Figure 6b,d,f. While the capacitance of the only GO-based sensors did not present significant changes for high values of RH (>75%), as already reported in previous works [13], the presence of PEDOT:PSS led to a considerable increase of the capacitance in this region. This effect was a consequence of the water molecules absorbed by the PSS, which formed a water meniscus layer once the saturation point was achieved [42]. In addition, both equivalent resistance and capacitance decreased when the frequency was increased as a result of the boost of the dielectric losses in the sensitive layer, mostly linked to the effect of the frequency on the dielectric constant of the GO [46]. This effect caused, on one hand, a reduction in equivalent capacitance between electrodes and, on the other hand, an increase in leakage current between them [47].

We summarize in Table 3 the results of both equivalent resistance at medium RH (50%) and sensitivity obtained for the different sensors at two different frequencies (100 Hz and 1 kHz). Furthermore, the sensitivity of the sensors as a function of the frequency can be found in Figure S4 (Supplementary Materials). It is important to note that the layout with thinner fingers actually helped to increase the parallel resistance of the sensors, but minimally modified their sensitivity. Moreover, as occurred with the capacitance of the sensors, in all cases, their sensitivity decreased as the frequency increased, due to the decrease in dielectric constant for both GO and PEDOT:PSS with respect to increasing frequency [46,48]. Furthermore, the highest sensitivity was obtained for an intermediate concentration of PEDOT:PSS since, although it helped to improve the sensitivity at higher RH values, if the concentration was too high, the performance of the sensors at low RH values worsened (see Figure 6), resulting in an reduction of the overall sensitivity. However, this fact changed when the frequency was increased above 10 kHz since, in that case, the sensitivity of the sensors increased with the concentration of PEDOT:PSS (as illustrated in Figure S4, Supplementary Materials). This was due to the fact that, above 10 kHz, the change in dielectric constant of GO and, therefore, its effect on the capacitance vanished rapidly for increasing frequencies and, consequently, the sensitivity relied mainly on the PEDOT:PSS.

Based on these results, the sensor which exhibited a better performance in terms of equivalent parallel resistance and sensitivity was that obtained with layout 2 (*W* = 115 µm, *i* = 225 µm) using GO/PEDOT:PSS (10%) as the sensitive layer. This sensitivity was more than four orders of magnitude higher than that one reported with an Ag-printed IDE structure with almost 10 times more area using polyimide as sensing layer (at the same frequency) [44], as well as much higher or comparing well with other similar sensors presented in the literature, as depicted in Table 4. 

**Figure 7:**
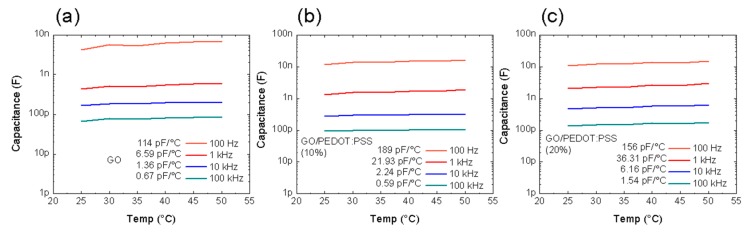
Effect of the thermal drift in the capacitance for the sensor with layout 2 (*W* = 115 µm, *i* = 225 µm) at different frequencies for the GO (**a**), GO/PEDOT:PSS (10%) (**b**), and GO/PEDOT:PSS (20%) (**c**) sensitive layers.

Moreover, the thermal drift of the sensors, i.e., their sensitivity to temperature variations, is also an important parameter. Therefore, we measured the dependence of the capacitance of the sensors with layout 2 as a function of the temperature for an intermediate value of RH (40%). The results obtained for the three sensitive layers are shown in Figure 7.

As seen, the capacitance was quite stable to temperature variations. In all cases, it increased as the temperature increased as a consequence of a slight increase in the dielectric constant. This effect was attributed to the highly oxidized nature of the sensitive layers and the effect of the temperature in the charge transfer from the carbon atoms to the oxygen atoms [53]. The thermal drift also decreased with increasing frequency since the changes in the dielectric constant of GO became negligible at high frequencies [54]. Nevertheless, the thermal drift of the sensors did not compromise their performance since, e.g., for the GO/PEDOT:PSS (10%) film, the thermal drift supposed less than 10% of the sensitivity value for any frequency. 

**Figure 8:**
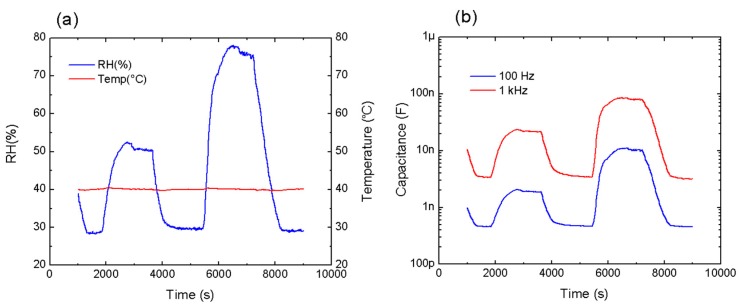
Transient response of the GO/PEDOT:PSS (10%) sensitive layer (*W* = 115 µm, *i* = 225 µm). (**a**) Values of temperature and RH obtained from the sensor incorporated in the climate chamber over time. (**b**) Capacitance response of the sensor at two different frequencies over time.

The response time of the sensors was also studied. GO was already demonstrated as a ultrafast material for humidity sensing [11]; therefore, we analyzed the sensor with the best overall performance (GO/PEDOT:PSS (10%) composite on layout 2) in order to assure that the GO/PEDOT:PSS-based sensors also provide good response and recovery times. For that, this sensor was tested applying two steps of different RH values (50% RH and 75% RH), as depicted in Figure 8. 

On one hand, Figure 8a shows both temperature and relative humidity changes in the climate chamber itself. As seen, the chamber needed a certain time to increase and decrease the RH. For this RH profile, the climate chamber presented the following response times: t_30%RH to 50%RH_ = 544 s, t_50%RH to 30%RH_ = 391 s, t_30%RH to 75%RH_ = 562 s, and t_75%RH to 30%RH_ = 752 s. Hence, the response time of the climate chamber was pretty stable for the humidification process; however, the dehumidification took quite longer depending on the RH range. We measured the time response of our sensors below this same RH profile, and the times obtained at a frequency of 1 kHz were as follows: t_30%RH to 50%RH_ = 610 s, t_50%RH to 30%RH_ = 398 s, t_30%RH to 75%RH_ = 701 s, and t_75%RH to 30%RH_ = 496 s. Then, our capacitive sensor had a time response around 10% slower than that incorporated in the climate chamber and a similar recovery time for low values of RH, where the sensitive mechanism was mainly associated with the GO. At low RH, the response of the sensor was faster since the water molecules were physisorbed onto the available active sites (hydrophilic groups and vacancies) of the GO surface, without penetrating the GO layers. However, at high RH levels, the increase in both capacitance and sensitivity occurred due to the permeation of the water molecules within the GO layers, as well as due to their interaction with the PEDOT:PSS chains, which took a longer time [11,55]. For that, the response was ~25% slower than that obtained with the sensor incorporated in the climate chamber for high values of RH. Furthermore, it can also be noticed that our sensor presented a recovery time faster than its response time in the whole range of RH indicating that the time of the desorption process was faster than required for the adsorption process [56].

**Figure 9:**
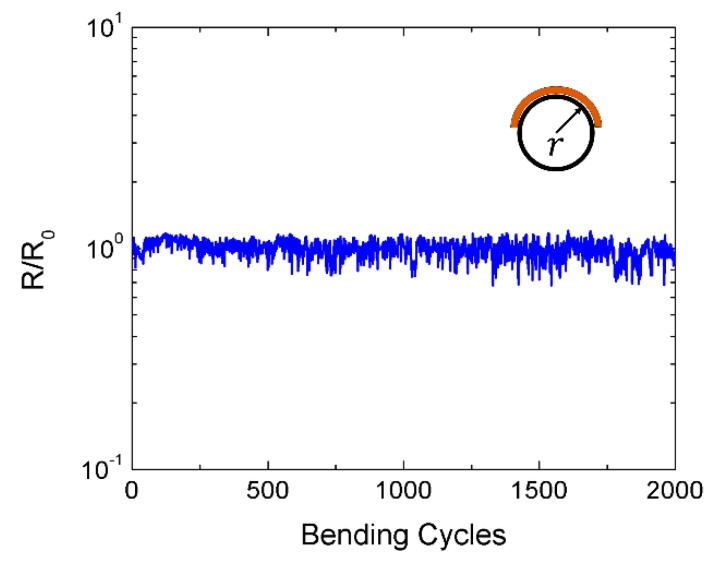
Change in resistance *R* with respect to the initial resistance *R_0_* for an increasing number of bending cycles. Inset diagram depicts the definition of bending radius (*r)*.

Finally, we further studied the changes in resistance of this latter sensor under mechanical stress. For that, the flexibility of the presented devices was analyzed for subsequent bending cycles with a minimum bending radius of 1.5 mm (see Figure 9). Thus, it can be recognized that, even after 2000 bending cycles, the change in the normalized resistance was below 15%, which indicated that the presented sensors also exhibit good stability and reversibility under bending cycles.

## 4. Conclusions

In summary, we reported the fabrication of thin-film capacitive sensors through the screen-printing of Ag-based planar IDE structures on flexible substrates. Using both GO and GO/PEDOT:PSS composites as sensitive layers, we studied the performance of the capacitors as humidity sensors. The results showed that the presence of the PEDOT:PSS within the GO structure was able to modify the electrical properties of the sensitive film, improving the overall performance of the sensors. We studied different IDE structures, as well as the influence of the PEDOT:PSS concentration on the sensitive layer. The experiments using hybrid GO/PEDOT:PSS composites as sensitive layer showed promising results regarding the increase in sensitivity to humidity changes when compared with other similar capacitive sensors from the literature as a consequence of the combination of the active region of both GO and PEDOT:PSS materials. The authors believe that this technology is a big step forward in the cost-effective fabrication of high-performance small flexible sensors, which could be expanded to a wide range of applications.

## Figures and Tables

**Table 1:** Planar IDE capacitor layout description.

Parameter	Layout 1	Layout 2
*W* (µm)	200	115
*i* (µm)	200	225
*S* (µm)	250	250
*L* (mm)	4.6	4.55
2 × N	12	14

**Table 2:** Experimental dimensions of the planar IDE capacitors.

Parameter	Layout 1	Layout 2
*W* (µm)	197.81 ± 13.12	148.54 ± 14.59
*i* (µm)	201.76 ± 8.53	233.55 ± 22.68
*S* (µm)	260.01 ± 35.88	277.15 ± 25.26

**Table 3:** Comparison of the equivalent parallel resistance and capacitance among the flexible capacitive humidity sensors presented in this work.

Figure of Merit	Sensing Layer	Layout 1 (*W* = 200 µm, *i* = 200 µm)	Layout 2 (*W* = 115 µm, *i* = 225 µm)
Equivalent parallel resistance (kΩ) at 50% RH	GO	28.11 at 100 Hz	93.09 at 100 Hz
		20.06 at 1 kHz	60.28 at 1 kHz
	GO/PEDOT:PSS (10%)	55.97 at 100 Hz	113.21 at 100 Hz
		27.35 at 1 kHz	60.76 at 1 kHz
	GO/PEDOT:PSS (20%)	90.45 at 100 Hz	84.22 at 100 Hz
		61.43 at 1 kHz	59.72 at 1 kHz
Sensitivity (nF/%RH)	GO	2.39 at 100 Hz	2.09 at 100 Hz
		0.21 at 1 kHz	0.23 at 1 kHz
	GO/PEDOT:PSS (10%)	10.45 at 100 Hz	11.53 at 100 Hz
		1.06 at 1 kHz	1.22 at 1 kHz
	GO/PEDOT:PSS (20%)	6.78 at 100 Hz	4.81 at 100 Hz
		0.49 at 1 kHz	0.37 at 1 kHz

**Table 4:** Comparison among related sensors presented in the literature. CNT—carbon nanotube.

Sensing Material	Sensitivity (pF/%RH)	Area (mm^2^)	Reference
GO/PEDOT:PSS	1220@1kHz	27	This work
Polyimide (PI)	0.025@1kHz	270	[44]
Cellulose acetate butyrate (CAB)	0.0023@100kHz	64	[49]
Processed PI	144.2@1kHz	100	[50]
Multi-walled CNTs/PI	0.65@20kHz	0.625	[51]
Reduced GO/SnO_2_	1604@10kHz	25	[52]
GO	46.25@1kHz	–	[12]

## Supplementary Materials

The following are available online at https://www.mdpi.com/2072-666X/11/2/148/s1, Figure S1: Actual view of one of the flexible RH sensors presented in this work, Figure S2: Absolute value of the impedance as a function of the relative humidity measured at different frequencies for both layout 1 (10 kHz (a) and 100 kHz (c)) and layout 2 (10 kHz (b) and 100 kHz (d)) using GO and the hybrid GO/PEDOT:PSS composites as sensitive layers, Figure S3: Equivalent parallel resistance and capacitance for layout 1 (*W* = 115 µm, *i* = 225 µm) at different frequencies using GO and the hybrid GO/PEDOT:PSS composites as sensitive layers, Figure S4: Sensitivity as a function of the frequency for the two layouts considered in this work as well as the three different sensitive layer.
