# Supplementary material for: Fabrication and Characterization of Humidity Sensors Based on Graphene Oxide–PEDOT:PSS Composites on a Flexible Substrate
Source: Micromachines (Basel). 2020 Jan 29;11(2):148. doi: 10.3390/mi11020148 (PMC7074611; doi:10.3390/mi11020148)
Supplement: Supplementary file 1 [file micromachines-11-00148-s001.pdf]

micromachines

MDPI

# Supplementary Material: Fabrication and Characterization of Humidity Sensors based on Graphene Oxide–PEDOT:PSS Composites on a Flexible Substrate

**Francisco J. Romero, Almudena Rivadeneyra, Markus Becherer, Diego P. Morales and Noel Rodríguez**

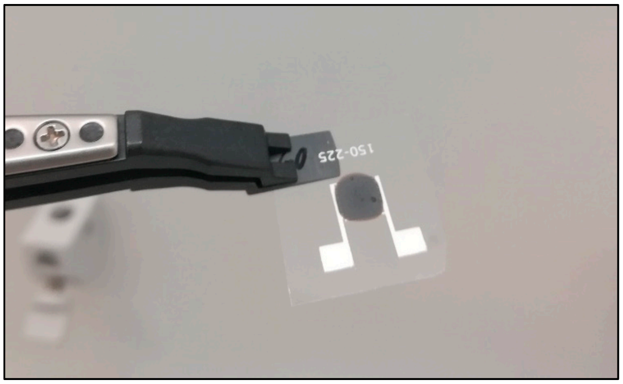

**Figure S1.** Actual view of one of the flexible RH sensors presented in this work.

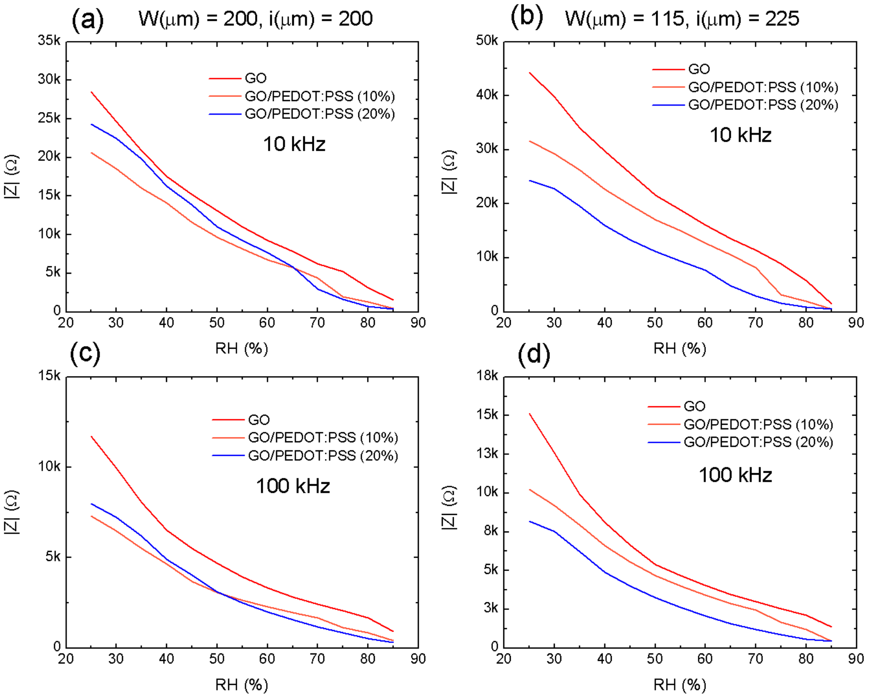


**Figure S2.** Absolute value of the impedance as a function of the relative humidity measured at different frequencies for both layout 1 (10 kHz (**a**) and 100 kHz (**c**)) and layout 2 (10 kHz (**b**) and 100 kHz (**d**)) using GO and the hybrid GO/PEDOT:PSS composites as sensitive layers.

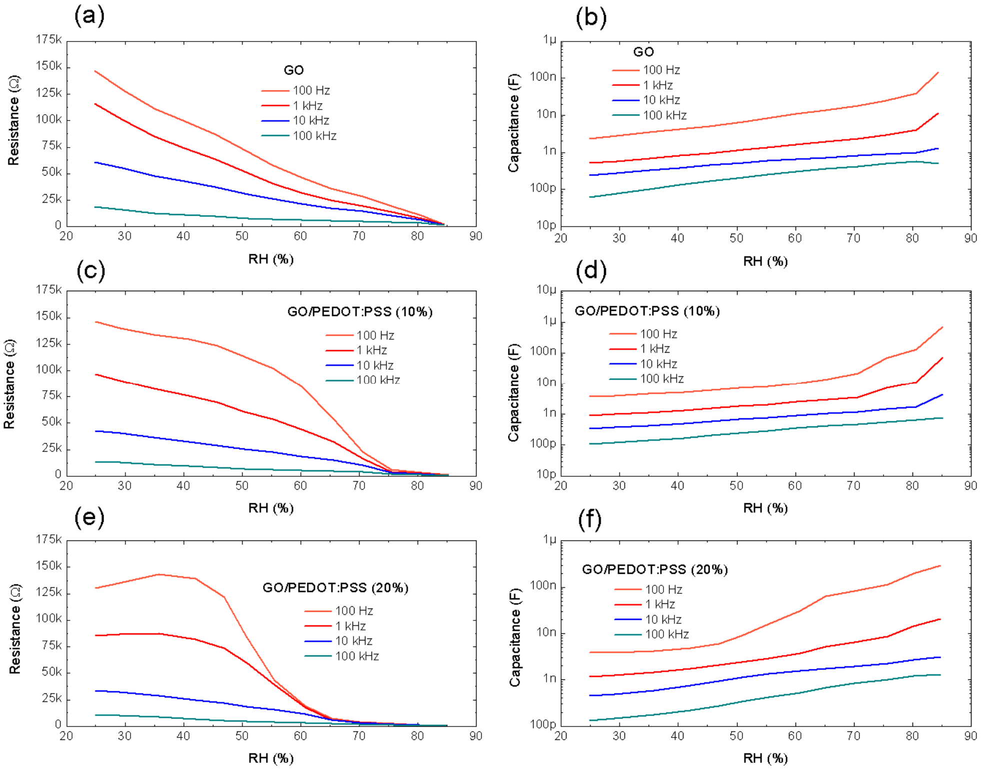


**Figure S3.** Equivalent parallel resistance and capacitance for layout 2 ($W$ = 115 µm, $i$ = 225 µm) at different frequencies using GO and the hybrid GO/PEDOT:PSS composites as sensitive layers; being (**a**) and (**b**) the results obtained for the GO layer, while (**c**)-(**d**) and (**e**)-(**f**) are the results associated to the GO/PEDOT:PSS (10%) and GO/PEDOT:PSS (20%) layers, respectively.

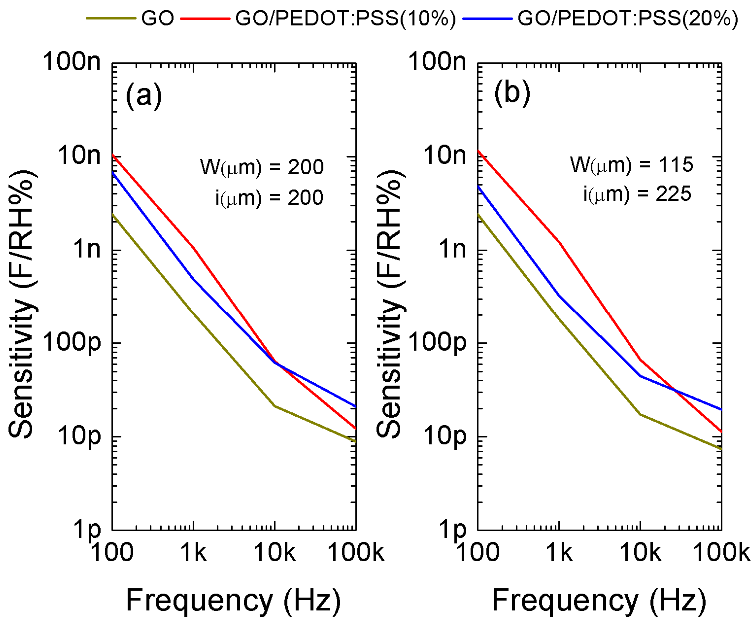


**Figure S4.** Sensitivity as a function of the frequency for the two layouts considered in this work ((**a**) layout 1, (**b**) layout 2), as well as the three different sensitive layer.
